# Structures of native human thymidine phosphorylase and in complex with 5-iodouracil

**DOI:** 10.1016/j.bbrc.2009.06.104

**Published:** 2009-09-04

**Authors:** Eirini Mitsiki, Anastassios C. Papageorgiou, Shalini Iyer, Nethaji Thiyagarajan, Steven H. Prior, Darrell Sleep, Chris Finnis, K. Ravi Acharya

**Affiliations:** a Department of Biology and Biochemistry, University of Bath, Claverton Down, Bath BA2 7AY, UK; b Novozymes Biopharma UK Ltd, Castle Court, Nottingham NG7 1FD, UK

**Keywords:** Thymidine phosphorylase, Crystal structure, Enzyme kinetics, Mutagenesis, 5-Iodouracil

## Abstract

Thymidine phosphorylase (TP) first identified as platelet derived endothelial cell growth factor (PD-ECGF) plays a key role in nucleoside metabolism. Human TP (hTP) is implicated in angiogenesis and is overexpressed in several solid tumors. Here, we report the crystal structures of recombinant hTP and its complex with a substrate 5-iodouracil (5IUR) at 3.0 and 2.5 Å, respectively. In addition, we provide information on the role of specific residues in the enzymatic activity of hTP through mutagenesis and kinetic studies.

Human thymidine phosphorylase (hTP, EC 2.4.2.4), identified as the sole endothelial mitogenic and angiogenic factor, belongs to the pyrimidine nucleoside phosphorylase (PyNP) family. In the presence of inorganic phosphate, it catalyzes the reversible cleavage of the glycosidic bond of pyrimidine 2′-deoxynucleotides, most likely through SN2-like transition state involving nucleobase, 2′-deoxyribose and phosphate. hTP has been found to play a role in the stimulation of chemotaxis and [^3^H] thymidine incorporation into endothelial cells *in vitro* and angiogenesis *in vivo*. hTP, functionally active as a homodimer plays a key role in the pyrimidine nucleoside metabolism, ensuring a sufficient pool of pyrimidine nucleotides is available for DNA repair and replication (for a detailed review see [1]).

Thymidine homeostasis is of great importance. Imbalances caused by elevated thymidine concentrations can alter cell growth and viability. hTP regulates thymidine concentration from reaching levels that would be inhibitory to endothelial cell proliferation. Presence of hTP in primary tumors is considered to be a risk factor leading to aggressive tumor progression, tumor growth and metastasis while its over-expression has been associated with angioproliferative disorders, such as rheumatoid arthritis and psoriasis [2]. The enzymatic activity of hTP has been shown to be indispensable for its angiogenic activity [3]. Modulating hTP activity could reduce tumor growth and metastasis as tumors depend on the salvage pathway for their proliferation. Its role in catalyzing the conversion of certain prodrugs, such as capecitabine, to anticancer nucleotides is of great significance as it directly interferes with DNA replication [4,5]. Given these observations, the potential for developing potent hTP inhibitors as therapeutic agents for cancer treatment and as anti-angiogenic agents is becoming apparent. A number of inhibitors have been designed to date and tested for their efficacy in inhibiting hTP, mostly through kinetic studies. Current inhibitors are mainly substrate analogues, while some purine-based inhibitors and synthetic inhibitors have also been reported [2].

Known PyNP structures to date include enzymes from *Escherichia coli*: uridine phosphorylase (EcUDP) and thymidine phosphorylase (EcTP), both in native and bound forms [6–11], the enzyme from *Bacillus stearothermophillus* (BsPYNP; [12]), and lastly the structure of hTP was reported in complex with a small molecule inhibitor [13] and in complex with thymine [14]. Insights gained from these structures suggest that a conformational change upon substrate (or inhibitor) binding appears to be essential for the phosphorolysis reaction to occur. In the two reported structures hTP both have the active site occupied, suggesting that the enzyme is in its closed–active conformation. Here, we report the crystal structures of recombinant native hTP and in complex with 5-iodouracil (5IUR), a substrate with a *K*_i_ of 0.48 mM. The structural work presented here was complemented with mutational and kinetic studies in order to comprehend the significance of the residues lining the active site of hTP.

## Materials and methods

*Expression and purification of recombinant native hTP and mutants*. cDNA clone for hTP was obtained from Mammalian Gene Collection. Stratagene QuikChange site-directed mutagenesis kit was used to introduce the desired mutations. DNA sequencing confirmed the presence of required mutations. *Escherichia coli* BL21-Codon-Plus-(DE3)-RIL cells harboring the recombinant plasmid of either native hTP or mutants were used to inoculate overnight cultures. Expression of the proteins in TB media was induced by the addition of 1 mM isopropyl β-d-thiogalactoside. Cells were incubated 3 h post-induction and then harvested by centrifugation. Cells lysis was carried out in a buffer containing 20 mM Na-citrate pH 5.7, 100 mM NaCl and EDTA-free protease inhibitor followed by the addition of lysozyme and mechanical disruption. The lysed cells were then subjected to centrifugation at 97,000*g* for 1 h at 4 °C. The clarified cell lysate (native or mutant hTP) was loaded onto a 5-ml Ni^2+^ His-select column (Sigma) previously equilibrated with buffer A (20 mM Na-citrate pH 5.7). Sample was loaded and the column was washed with buffer A before eluting the protein using an imidazole gradient (0–250 mM). Fractions were analyzed on a 15% SDS–PAGE and those containing significant amounts of hTP were pooled. Final purification was carried out by gel filtration in buffer C (20 mM Na-citrate pH 5.7, 100 mM NaCl). Eluates were analyzed on a 15% SDS–PAGE to check purity of the protein before pooling. Concentration of hTP and mutants was determined using the BCA assay method (standard BCA Assay, Pierce). Absorbance was measured at 562 nm. The concentration of hTP was ∼8.8 mg/ml and that of the various hTP mutants ranged from 7.5 to 20 mg/ml.

*X-ray crystallography*. Crystals for hTP and hTP–5IUR complex were obtained by the hanging drop vapor diffusion method. Crystals of hTP grown in 0.1 M Na-acetate (pH 5.2), 25% PEG 4000 and 0.1 M ammonium acetate belong to P2_1_ space group. Crystals for the hTP–5IUR complex were obtained after overnight incubation of hTP with 100 mM 5IUR at 4 °C prior to equilibration with reservoir solution containing 2.8–3.5 M Na-formate (pH 8.7) at 16 °C in P2_1_2_1_2_1_ space group. All datasets were collected at 100 K in the presence of 25% glycerol as cryoprotectant. Both structures were solved by molecular replacement method using the program MOLREP [15]. PDB code 1UOU [13] was used as search model for hTP–5IUR complex. The final refined structure of the complex has a crystallographic *R*-factor (*R*_cryst_) of 22.7% and free *R*-factor (*R*_free_) of 28.3% (Table 1). hTP from the complex was used to solve the structure of native hTP. The structure was refined to an *R*_cryst_ of 20.7% and an *R*_free_ of 28.4% (Table 1) using CNS [16].

*Activity assays and inhibition of hTP by 5IUR*. Enzymatic activity of recombinant and mutant hTP was assayed by the spectrophotometric method [17]. Purified enzyme (native and mutants) was prepared to a final molarity of 40 nM and was incubated with 0.1 M MES pH 5.5, 0.1 M KH_2_PO_4_ pH 5.5 and thymidine (Sigma) in a total reaction volume of 100 μl. Substrate concentrations tested ranged from 200 μM to 1.2 mM. Reactions were incubated at 37 °C and stopped by the addition of 900 μl of 0.1 M NaOH. Thymine production was measured by absorbance at 300 nm, where the difference in the extinction coefficient between thymine and thymidine is 3400 M^−1^ cm^−1^. Assay results were analyzed with GraFit (Erithacus Software; [18]) for obtaining the *K*_m_, *V*_max_ and standard error values. *V*_max_ values were then used to calculate *k*_cat_.

Enzymatic activity assays for native hTP in the presence of 5IUR were carried out in order to determine its potency as an inhibitor. Previous kinetic studies carried out by Razzell and Casshyap [19] have shown a 61% inhibition of thymidine arsenolysis when 0.64 mM of 5IUR was used. Concentration of native hTP was retained at 40 nM and the substrate concentrations were as previously described (200 μM to 1.2 mM). Concentrations of 5IUR were 0.005, 0.01, 0.05, 0.1, 0.15 and 0.2 mM. Assay results were used to generate Dixon plots for the determination of *K*_i_ for 5IUR [20].

## Results and discussion

### Details of native and 5IUR complex structures

**Fig. 1:**
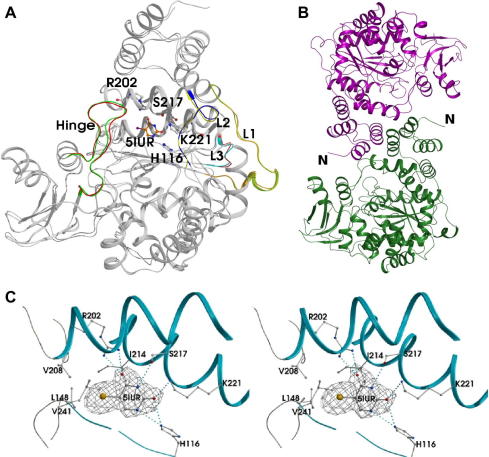
(A) Superposition of hTP molecule in the native (gray) and in complex with 5IUR (light orange). Highlighted in green are the three loops (L1–L3) and in magenta is the hinge region. (B) Crystal packing of hTP–5IUR complex. The figures were created using Pymol (www.pymol.org). (C) Stereo view of the active site with bound 5IUR in the hTP–5IUR complex. Potential hydrogen bonds are illustrated with dotted lines. The electron density map (2*F*_o_ − *F*_c_) around 5IUR is contoured at 1.0σ level. The figure was created using Molscript [24].

The crystal structure of native hTP (at 3.0 Å) (Fig. 1A) consists of two non-crystallographic dimers in the asymmetric unit. All four copies of hTP contain residues 34–479, residues 32 and 33 can be observed for chains A and B while only residue 33 can be observed for molecule C. At the C-terminal end, residue 480 was observed only in chain B. The crystal structure of hTP–5IUR complex (at 2.5 Å) consists of a dimer per asymmetric unit (Fig. 1B). Both chains comprise of residues 35–480 with one molecule of 5IUR bound in the active site of each hTP molecule.

No secondary structure differences were observed between native hTP, hTP–5IUR complex structure and the previously reported hTP structures [13,14]. The active site is located at the interface between the α- and the mixed α/β-domain (Fig. 1A). The N-terminal helices of the two monomers form the interface and bury a total surface area of around 1289 Å^2^ upon dimerization.

### Phosphate binding and domain movement: native hTP against hTP–5IUR complex

Of the two substrates that are required for catalysis by hTP, phosphate is considered to bind first, followed by the binding of thymidine. The phosphate-binding site is proposed to be located near the active site cleft, between strands β1 and β2 (Fig. 1A; [12]). Domain movement (once the active site is occupied) is considered to be essential in bringing the substrates into close proximity for the reaction to proceed and has been proposed for other members of the PyNP superfamily as well. Such a movement depending on the bound and unbound state of the enzyme, has also been supported based on computational work on EcTP [21]. Structural alignment of bound (1TPT) and unbound (2TPT) EcTP yielded an rmsd of 0.52 Å for 440 Cα atoms.

Pugmire and Ealick [12] have also suggested that phosphate-binding leads to the formation of a key hydrogen bond between the side-chain of His116 and the carbonyl oxygen of Gly205 (corresponding residues His150 and Ala239 in hTP), resulting in the rotation of the α/β-domain with respect to the α-domain by 8°. Subsequent binding of pyrimidine in the active site cleft is thought to cause a further 20° rotation thereby leading to a fully closed conformation [12]. So far the two reported crystal structures of hTP were both in the closed form [13,14], based on which we can argue that binding of 5IUR in the active site of hTP leads to closed conformation.

The crystal structure of unbound hTP provided some interesting findings. Alignment of the molecule A of the two structures (hTP and hTP–5IUR complex) indicates that they align well (including the flexible loop regions and the hinge region; Fig. 1A), with an rmsd of 0.57 Å over 445 Cα atoms. The ‘key hydrogen bond’ between His150 and Ala239 previously proposed to form the phosphate-binding site is present in both bound and unbound hTP structures. Structural alignment of BsPyNP (1BRW), hTP–5IUR complex and native hTP revealed no conformational change, although the ‘key hydrogen bond’ was detected in all cases. Hence, it is unlikely that only the binding of phosphate would trigger the formation of this key hydrogen bond as it is also observed in structures where a water molecule is present instead of a phosphate ion at the phosphate-binding site.

Previous studies propose that hTP undergoes a nucleophilic transition state of an S_N_2-type, the first ever to be observed for the family of *N*-ribosyl transferases [1]. Since no structure is available for the transition states of hTP, it is not possible to say conclusively whether any conformational change occurs during catalysis. It is evident that presence of substrate analogue is not required for the enzyme to acquire the ‘closed’ conformation. However, it is possible that the closed conformation observed for both bound and unbound hTP is favoured by crystal packing, while different ‘conformation/s’ of the enzyme may exist in solution.

### Comparison of hTP with other known TP structures

hTP, EcTP and BsPyNP share an overall sequence similarity of ∼42%. Most of the α-domain and part of the mixed α/β-domain are conserved. The active site residues are conserved among the three PyNPs. The only exception is Val241 in hTP, which corresponds to Phe207 in BsPyNP and Phe210 in EcTP. Overall structural comparison of hTP (present structures and the two previously reported, i.e., 1UOU, 2J0F), EcTP [1TPT, 2TPT] and BsPyNP (1BRW)] gave an insight into the topological similarity of the three PyNPs (Table 2).

The glycine rich loop described in EcTP [22] is also present in BsPyNP and hTP (residues 144 to 154 in hTP). The loop, preceded by a β-strand (β2) and followed by a α-helix (α6), was proposed to have a role in the binding of the catalytic phosphate [22]. In our structure, however, the phosphate-binding site was occupied by a water molecule and hence the significance of this glycine rich loop, if any, for the binding of phosphate could not be determined.

### Interactions of hTP residues with 5IUR

The active site of hTP consists of residues His116, Ser117, Leu148, Arg202, Val208, Ile214, Lys221 and Val241. A total of six potential hydrogen bonds stabilize the binding of 5IUR in the active site of hTP (Table 3). His116 directly interacts with 5IUR via its NE2 group. Almost opposite to His116 is Arg202, bonded via its amino groups to O4 of 5IUR. The other residues which are also involved in hydrogen-bonding interactions with 5IUR are Ser217 and Lys221 (Fig. 1C and Table 3).

Eleven hTP residues are involved in potential van der Waals interactions with 5IUR [13] have suggested that Val208 and Ile214 of the α-domain and Leu148 and Val241 of the α/β-domain stabilize the chlorine atom of TPI by forming a hydrophobic pocket. The iodine atom of 5IUR, appears to be similarly stabilized in the hydrophobic pocket (Fig. 1C).

### Correlation of structural data with results from mutagenesis experiments

Structural analysis on members of the PYNP superfamily have revealed several key residues that are conserved among species. Conserved residues of particular interest are those that are involved in the binding and stabilization of the substrate/inhibitor in the active site cleft. Using the structural information gained from the hTP–5IUR complex, a series of mutations were designed for several amino acids that were seen to line the active site of the enzyme. Two of the mutants, K115E and R202S, previously reported by Miyadera et al. [23], were repeated to act as controls (along with native hTP) for the rest of the mutations. Biological activity of hTP was adjudged by an enzymatic activity assay. The assay revealed that the native hTP was catalytically active with a *k*_cat_ of 8.2 s^−1^. Of the mutations on Lys115, His116, Tyr199, Arg202, Ile214 and Ser217, only four yielded some degree of enzymatic activity (Table 4). As expected the previously characterized mutants (Lys115Glu and Arg202Ser) were inept at catalyzing the breakdown of thymidine to thymine.

His116 is a residue of key importance in PyNPs. Previous studies on the equivalent residue of EcTP (His85) by means of targeted molecular dynamics suggest that this highly conserved residue might be important for the catalytic mechanism of TP [42]. Norman et al. [13] have also discussed its participation in a proton shuttling mechanism, where Asp114, Glu225 and Lys222 form a triad delivering a proton to His116. Mutation of His116 to a phenylalanine or a lysine has a severe effect on hTP as both mutations completely abolish the enzymatic activity. It is likely that the basic charge and the ring structure of histidine at this position are an absolute requirement for the catalytic activity.

hTP amino acid sequence possess only one tyrosine residue when compared to other members of the PYNP superfamily. It is quite interesting to note that this sole tyrosine residue is located at the active site cleft, although it does not form any hydrogen bond with the inhibitor. Among the different tyrosine mutations tested, Tyr199Leu and Tyr199Phe reduced the catalytic activity of hTP, while Tyr199Ala completely abolished it (Table 4). It is possible that both size as well as polar character of the side chain by virtue of its hydroxyl group is essential for maintaining the structural integrity of the catalytic site.

Along with the previously characterized Arg202, also involved in the network of interactions that stabilize 5IUR in the active site cleft of hTP is Ser217. This residue was observed to be involved in reinforcing the molecules bound at the active site by means of hydrogen bonds [13]. In the present study, Ser217 was mutated to glycine and the mutant assayed for enzymatic activity. No thymine production could be detected, indicating that the mutation was detrimental to the enzymatic activity. The mutation corresponds to the loss of a potential hydrogen bond involving the hydroxyl group of the serine.

Apart from the residues that interact with 5IUR via hydrogen bonds, a hydrophobic pocket lined by Leu148, Val208, Ile214 and Val241, contributes to the stabilization of the molecule by holding the ‘iodine’ atom in place. This is similar to the hTP–TPI complex [13] where TPI is held in place in the hydrophobic pocket via its ‘chlorine’ atom. Mutational work reported for Leu148 (Lue148Arg) revealed the loss of enzymatic as well as angiogenic activity. In contrast, mutations on Ile214 (another residue contributing to the hydrophobic pocket) showed reduction in the catalytic efficacy of the enzyme. The mutations, however, did not abolish the enzymatic activity of hTP.

## Conclusion

Thymidine phosphorylase, providing pyrimidine nucleotides for DNA repair and replication, has also been shown to have angiogenic activity *in vivo*. Mutational study has established that the angiogenic activity of hTP is dependent on its enzymatic activity. As hTP is directly involved in angiogenesis, inhibition of its enzymatic activity is thought to be of key importance in the inhibition of angiogenesis, which in turn will lead to cessation of tumor growth and metastasis. Inhibitors of hTP can act as anti-angiogenic agents, significantly reducing tumor growth by disrupting the thymidine salvage pathway, angiogenesis or metastasis. Several inhibitors have been designed for hTP and have mostly been tested in kinetic studies. Only one crystal structure of hTP in complex with a small molecule inhibitor has been reported to date (1UOU; [13]). The present structures of native hTP and hTP–5IUR complex provide further information about the active site features of hTP. Site-directed mutagenesis and kinetic analyses on native and selected mutants of hTP have revealed the key role of Lys115, His116, Tyr199, Arg202, Ile214 and Ser217 in enzyme catalysis.

## Figures and Tables

**Table 1:** Crystallographic data.

	hTP–5IUR complex	hTP native
Space group	Orthorhombic P2_1_2_1_2_1_	Monoclinic P2_1_
Cell dimensions	*a* = 61.9, *b* = 67.3, *c* = 212.3 Å	*a* = 103.5, *b* = 77.2, *c* = 100.9 Å, *β* = 98.0°
Resolution range (Å)	50–2.5	50–2.97
*R*_symm_ (%)	5.3 (18.5)	13.8 (43.7)
*I*/*σI* (outer shell)	22.0 (5.9)	6.6 (1.7)
Completeness (outer shell) (%)	94.5 (69.6)	90.5 (39.6)
Reflections (total/unique)	319,417/33,589	108,957/33,291
Redundancy	4.4 (2.8)	2.3 (1.7)
Number of water molecules	44	19
*R*_cryst_ (%)	22.7	20.7
*R*_free_ (%)	28.3	28.4
RMSD in bond lengths (Å)	0.007	0.007
RMSD in bond angles (°)	1.3	1.4
Ramachandran plot statistics (%)	100	98.7
Average *B*-factor (Å^2^)	**A**: 38.8; **B**: 45.2 **5IUR(A)**: 36.8; **5IUR(B)**: 38.7	**A**: 22.7; **B**: 22.8; **C**: 34.7; **D**: 35.5

PDB codes	2WK6, 2WK6SF	2WK5, 2WK5SF

**Table 2:** Comparison of hTP with other known structures.

PDB codes	Root mean square deviation (rmsd) (Å)	
	hTP–5IUR	Native hTP
2J0F (hTP)	0.6 (445)	0.6 (446)
1UOU (hTP)	0.8 (436)	0.9 (437)
1TPT (EcTP; native)	3.3 (435)	3.3 (436)
2TPT (EcTP; complex)	3.2 (435)	3.2 (436)
1BRW (BsPyNP)_	2.9 (426)	3.0 (426)

The numbers in parentheses are the Cα atoms over which the rmsd values were calculated.

**Table 3:** Putative hydrogen bonds between hTP active site residues and 5IUR.

hTP atom	IUR atom	Distance (Å)	
Molecule A		Molecule A	Molecule B
His116 NE2	N1	2.9	2.7
His116 NE2	O2	3.0	3.1
Arg202 NH1	O4	2.9	3.3
Arg202 NH2	O4	2.7	3.0
Ser217 OG	N3	3.1	3.0
Lys221 NZ	O2	2.6	2.7

**Table 4:** *K*_m_, *V*_max_ and *k*_cat_ values for native hTP and hTP mutants.

	*K*_m_ (mM) (Std. error)	*V*_max_ (μM/min) (Std. error)	*k*_cat_ (s^−1^)
Native hTP	0.35 (0.01)	19.7 (2.0)	8.2
K115E	–	–	–
K115A	0.42 (0.04)	16.4 (0.9)	6.8
H116F	–	–	–
H116K	–	–	–
Y199A	–	–	–
Y199L	3.14 (1.0)	10.6 (2.0)	4.4
Y199F	2.1 (0.6)	8.4 (1.0)	3.5
R202E	–	–	–
R202S	–	–	–
I214A	0.74 (0.03)	12.53 (0.3)	5.2
S217G	–	–	–
